# Human and Animal Tuberculosis

**Published:** 1907-02-09

**Authors:** 


### The Hospital

A Journal of

The Medical Sciences and Hospital Administration.

Vol. XLI.—No. 1065. SATURDAY, FEBRUARY 9, 1907.

### Human and Animal Tuberculosis.

The study of tuberculosis during the last quarter of a century has produced dramatic surprises not a few, and among such unexpected developments must certainly be ranked Professor Koch's statement, made in 1900, that human and bovine tuberculosis were two distinct and separate diseases. Previous to that announcement it had been taken for granted that tubercle, wherever found, was one and the same disease, the result of one and the same bacillus, which might, so it was assumed, be readily transferred from the lower animals to man, or *vice versa*. Koch's view was received with general suspicion, or even with more definite hostility, both by the medical and veterinary professions. On the other hand, it was naturally welcomed by those financially interested in the public supply of meat and milk, for it suggested that certain restrictions on the sale of these articles were no longer necessary, the bacillus of bovine tuberculosis being, according to the new doctrine, incapable of infecting the human body. It soon became generally recognised that a large public issue was thus involved, and that an authoritative attempt must be made to settle the question. In response to this a Royal Commission was appointed, and this body has since been engaged in an extensive series of experimental investigations which have been conducted on farms generously placed at the disposal of the Commission by Sir James Blyth. In 1904 the Commission issued a first interim report, in which it was announced that the experimental work of the Commission showed that human tuberculosis could be communicated to bovine animals, and that generally, Koch's assumptions had little or no foundation, and could not safely be made the basis for conduct or legislation.

The Commission has now issued a second interim report, and in this the conclusions stated in the first report are confirmed and the results of further experimental work are presented. First may be noted the broad result of introducing into the bodies of animals of various natural orders tuberculous material taken in one group from cattle, and in another from human beings. When comparing these two series of cases the Commissioners record rapidly fatal results in all the animals injected, excepting only rats and mice, and state that the effects produced were the same both in kind and degree whether the material employed was taken from the bodies of tuberculous human beings or from tuberculous bovine animals. This is emphatic and definite enough, and it will be difficult any longer to obtain a hearing for the proposition that human and bovine tuberculosis are distinct diseases, and that one cannot possibly give rise to the other.

A further comparison is instituted between the two series of cases above referred to in reference to the microscopical and cultural characteristics of the respective bacilli. And here the Commissioners are equally confident. They deny that any differences between the bacilli in the two groups can be recognised, and, further, they contend that the disease, as seen in the human bodies examined, not only had all the features of bovine tuberculosis, but that the bacilli giving rise to the disease actually came from a bovine animal. "There can be no doubt," says the Report, "but that in a certain number of cases the tuberculosis occurring in the human subject, especially in children, is the direct result of the introduction into the human body of the bacillus of bovine tuberculosis." In equally decisive tones it is concluded that the bacillus is introduced through the agency of cow's milk, which, as a medium for the bacilli, is held to be responsible "for a considerable amount of disease and loss of life, especially among the young."

The immediately practical outcome of the work of the Commission needs no moral to enforce it. The effective supervision of dairy farms is a demand which can no longer be resisted, and this is the more necessary seeing that the clinical detection of tuberculosis in the cow is a much more certain task than the detection of the bacilli in the milk. It is at the source of supply that the evil must be suppressed. If this is recognised and enforced the public value of the Commission will need no further justification. There is, however, still much work to be done. In particular, it is obvious from the observations already made, that whilst the bacillus and the effects it produces are, each alike, identical in human and bovine tuberculosis, there are certain conditions and circumstances which modify the virulence of the bacilli.

The work in this direction is to be continued, and as it manifestly has therapeutic possibilities, it will command the special interest of the medical profession. The Commission has already accomplished much, and its further work will be followed with confidence and expectation.

